# A Case Report of Forehead Subgaleal Lipoma: Diagnostic Dilemmas and Surgical Solutions

**DOI:** 10.7759/cureus.50760

**Published:** 2023-12-19

**Authors:** Suresh R Chandak, Shruthi Bikkumalla, Meenakshi S Chandak

**Affiliations:** 1 General Surgery, Jawaharlal Nehru Medical College, Datta Meghe Institute of Higher Education and Research, Wardha, IND; 2 Dermatology, Jawaharlal Nehru Medical College, Datta Meghe Institute of Higher Education and Research, Wardha, IND

**Keywords:** surgical excision, ct scan, case report, benign soft tissue tumor, forehead, subgaleal lipoma

## Abstract

Subgaleal lipomas are an uncommon type of benign soft tissue tumour. They typically present as painless, slow-growing masses in the subcutaneous plane beneath the galea aponeurotica of the scalp. This case report presents a rare subgaleal lipoma in a 50-year-old female patient with a chief complaint of painful swelling on her forehead above the left eyebrow. Diagnostic imaging revealed a well-defined, round, fat-density mass in the subcutaneous plane of the bilateral frontal and high parietal midline region, causing a mass effect. The patient underwent successful excision of the subgaleal lipoma under local anaesthesia, leading to relief from the swelling and associated pain with no reported complications during the recovery period. Follow-up assessments confirmed the absence of recurrence.

## Introduction

Subgaleal lipomas are rare, benign soft tissue tumours that typically present as slow-growing, painless masses beneath the scalp's galea aponeurotica. Their subtle clinical appearance primarily distinguishes them and is often detected due to cosmetic concerns rather than any associated discomfort or pain [1].

In clinical practice, encounters with scalp masses are commonplace. Ultrasound is typically the initial imaging modality employed for assessing soft tissue masses in the scalp, primarily owing to its accessibility and cost-effectiveness. Diagnosing scalp lipomas using point-of-care ultrasound is straightforward due to their characteristic semi-spherical shape and delicate internal echogenic lines parallel to the tumour's longitudinal axis [2]. Subgaleal lipomas, in particular, are identified by their location between the galeal aponeurosis and the cranial bone, with most cases reported in the forehead region [3,4].

The precise epidemiological and demographic characteristics of intramuscular lipomas require further clarification. This lack of clarity is partly attributed to the relative rarity of intramuscular lipomas and their tendency to be grouped with other deep-seated and superficial lipomas in previous investigations. Such grouping has contributed to the challenges in establishing comprehensive epidemiological and demographic profiles for these tumours [5,6]. It is important to note that intramuscular lipomas constitute a specific form of lipomas, with some categorized as "atypical lipomas."

## Case presentation

**Figure 1:**
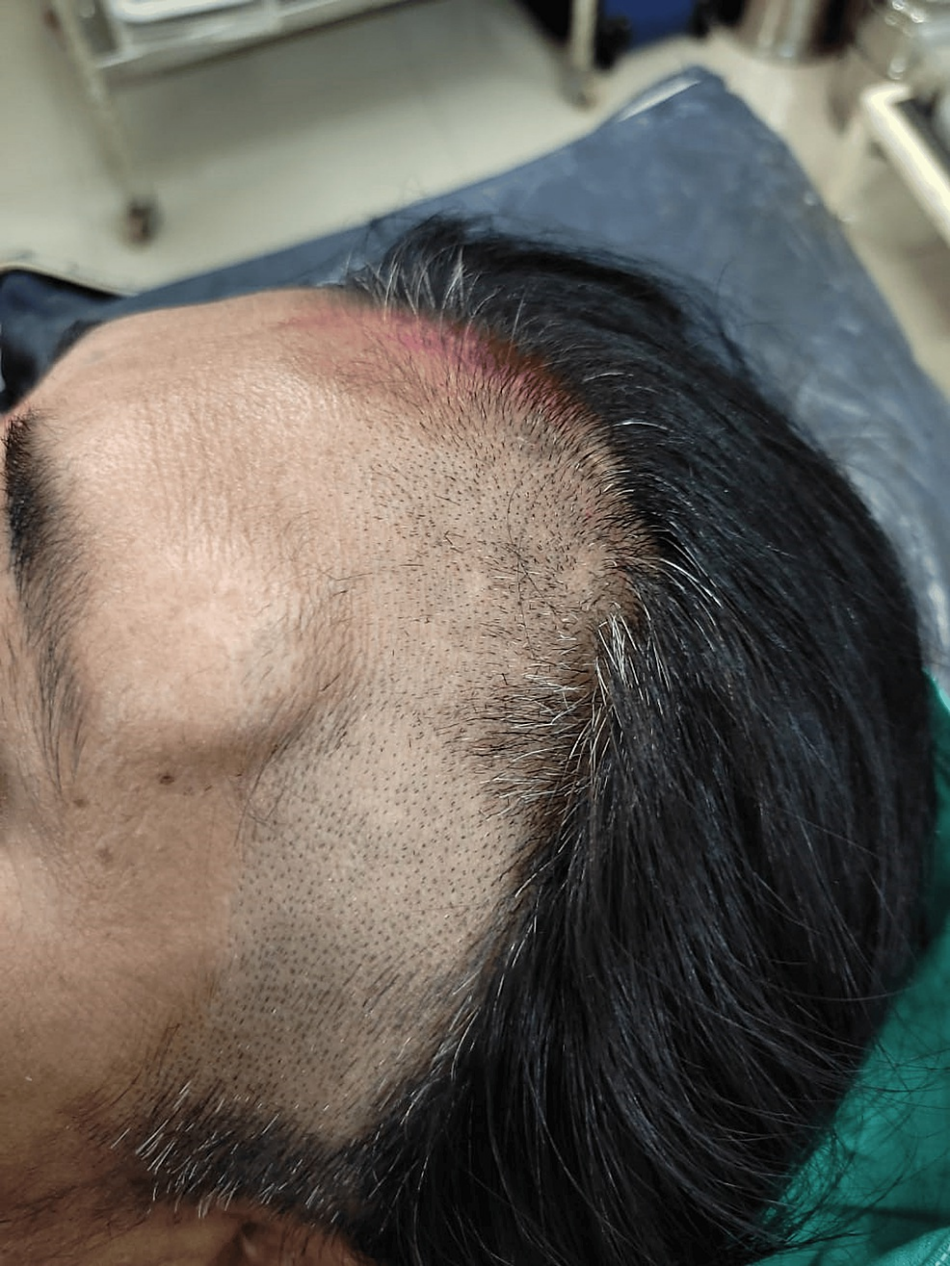
The figure shows the swelling on the forehead.

A 50-year-old woman in her middle years presented with a chief complaint of a noticeable swelling on her forehead that had been present for the past month, situated directly above the left eyebrow (Figure 1). A subgaleal lipoma was identified upon physical examination, characterized by a slow-growing, painless, firm, and immobile swelling on the frontal scalp. The dimensions of this swelling measured approximately 3 x 3 centimetres. Following a comprehensive patient history and examination, the differential diagnosis included sebaceous cysts, epidermoid cysts, dermoid cysts, trichilemmal cysts, intraosseous hemangiomas, and other vascular malformations. Malignant lesions such as lymphoma, carcinoma, and metastasis were also considered.

In addition to the swelling, the patient reported concurrent pain, which was exacerbated during physical activity but subsided upon cessation of movement. The examination revealed a solitary, palpable enlargement in the affected area.

**Figure 2:**
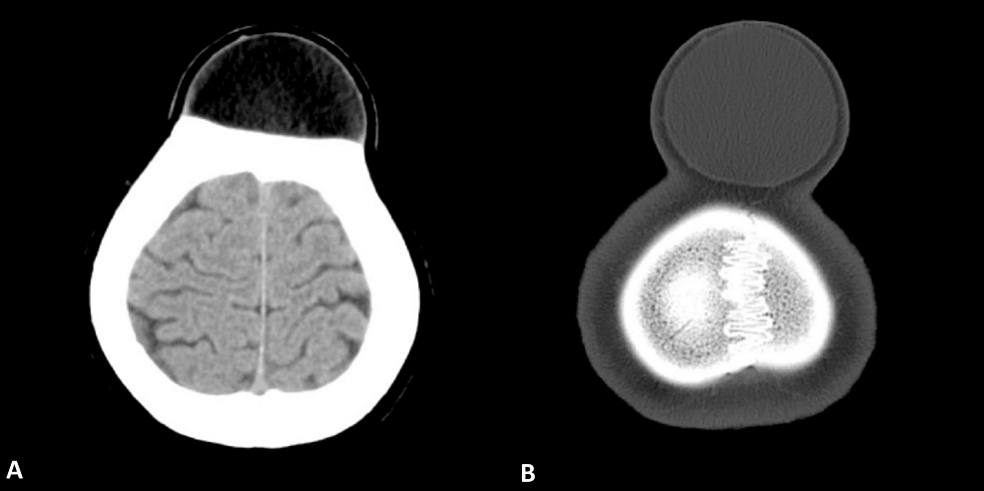
The computed tomography image shows (A) the fat density and (B) the entire lipoma.

Further investigation via a computed tomography (CT) scan unveiled a well-defined, round mass with a fat density ranging from -10 to 30 Hounsfield Units (HU). This mass was situated extra-axially within the subcutaneous plane of the bilateral frontal and high parietal midline region (Figure 2). Notably, it exerted a mass effect, causing a noticeable bulging of the bilateral frontal and parietal bones. These radiographic findings strongly suggested the presence of a subgaleal lipoma.

**Figure 3:**
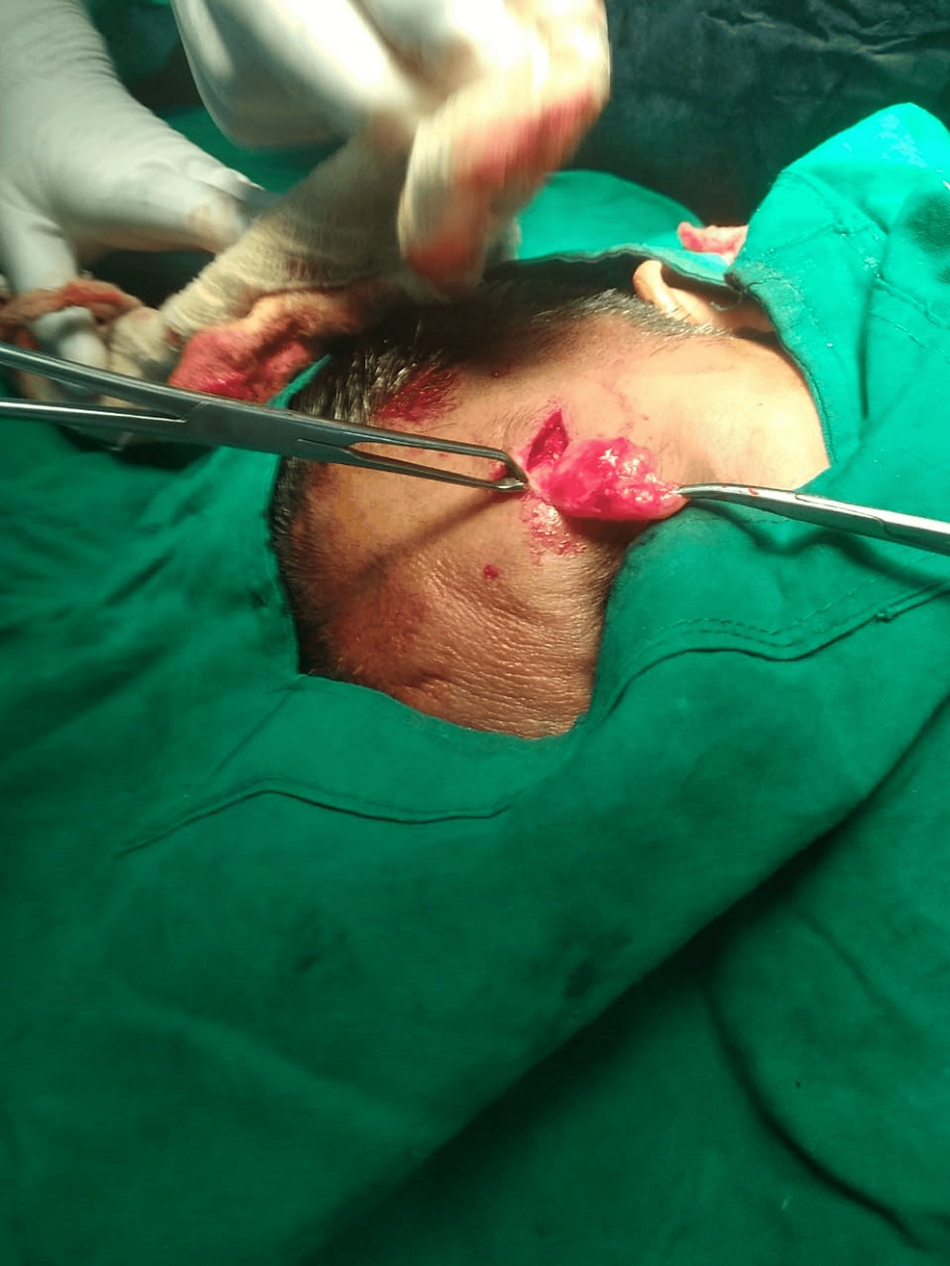
The figure shows the surgical excision of the subgaleal lipoma.

Following this, a surgical procedure was performed for the excision of the subgaleal lipoma under local anaesthesia, as illustrated in Figure 3. The surgical intervention was carried out meticulously to preserve the overlying scalp and minimize potential cosmetic consequences. Notably, during the intraoperative phase, it was observed that the lipoma was encapsulated and easily dissected from the surrounding tissues. Regarding the approach, consideration was given to maintaining an incision beneath the hairline to address both the successful excision of the lipoma and cosmetic concerns. Additionally, the anatomical location of the lipoma was confirmed to be subgaleal, beneath the galea aponeurotica.

The patient reported relief from swelling and associated pain after the surgical excision. No documented complications were observed throughout the 14-day postoperative recovery period. The patient was kept in the hospital for the entire recovery period due to residing in another state and being unable to afford travel expenses. This decision facilitated a thorough 14-day follow-up.

Subsequent assessments and examinations during follow-up consistently confirmed the absence of any recurrence of the lipoma. Notably, intramuscular and atypical lipomas, in particular, have been known to exhibit late recurrence, spanning from years to decades. This case highlights the successful management of an uncommon presentation of a subgaleal lipoma in the forehead region. The prompt diagnosis, followed by surgical intervention, resulted in favourable outcomes, with the patient experiencing relief from her symptoms and no complications during the recovery phase.

The absence of recurrence during follow-up underscores the importance of complete excision and vigilant postoperative monitoring in managing subgaleal lipomas.

## Discussion

A subgaleal lipoma is a heterotopic adipose tissue tumour typically found between the periosteum and the galea aponeurosis, also known as the epicranial aponeurosis, within the scalp [7]. In this case, CT imaging played a pivotal role in precisely characterizing the subgaleal lipoma. The CT scan revealed a well-defined, circular mass with the characteristic fat density range of -10 to 30 HU, situated in the subcutaneous layer of the forehead. The clear delineation of the tumour's borders and its observed impact on the adjacent frontal and parietal bones were consistent with the typical characteristics of subgaleal lipomas. It is essential to underscore that diagnostic imaging is instrumental in identifying and accurately characterizing such tumours, thereby facilitating the formulation of appropriate surgical plans [8].

Many cases of forehead lipomas are reported to be deep to the muscle layer, and ultrasound is used to delineate the depth of forehead lipomas [9]. The presentation of subgaleal lipoma can include a large frontal swelling with disfigurement, and patients may not necessarily experience pain, skin discolouration, or discharge [10]. The excision of the subgaleal lipoma was successfully performed under local anaesthesia. This choice of anaesthesia minimized patient discomfort and allowed for meticulous dissection of the encapsulated lipoma from surrounding tissues. Preservation of the overlying scalp was crucial to minimize cosmetic consequences. The surgical approach underscores the importance of careful planning and execution in achieving favourable outcomes for patients with subgaleal lipomas [11].

The atypical presence of pain associated with the subgaleal lipoma prompts consideration for a histological examination to explore the possibility of findings suggestive of an angiolipoma. In general, subgaleal lipomas are known to be painless, and their discovery is often prompted by cosmetic concerns rather than discomfort. The unique presentation of pain in this case may be attributed to various factors, such as compression of surrounding structures or potential nerve involvement. Further studies could delve into these aspects to provide a more comprehensive understanding [12].

Postoperatively, the patient experienced relief from the swelling and the associated pain. Notably, no complications were reported during the recovery period. The absence of recurrence during follow-up assessments emphasizes the importance of thorough surgical excision and diligent postoperative monitoring. Long-term follow-up is essential to ensure the continued well-being of patients with subgaleal lipomas [13]. This case report underscores the importance of considering atypical presentations of subgaleal lipomas in clinical practice. It serves as a reminder that such tumours can occasionally cause pain and discomfort, necessitating prompt diagnosis and management. Clinicians should know the potential variability in clinical presentation and the importance of tailored treatment approaches for individual cases.

## Conclusions

In conclusion, this case report highlights an atypical presentation of a subgaleal lipoma in the forehead region, wherein the patient experienced visible swelling and concomitant pain exacerbated by physical activity. The diagnostic evaluation, including a CT scan, played a crucial role in accurately characterizing the mass and guiding the surgical approach. The successful excision of the subgaleal lipoma under local anaesthesia led to the prompt resolution of the patient's symptoms, including relief from both the swelling and associated pain. Notably, the absence of any complications during the postoperative period and the confirmation of no recurrence during follow-up assessments underscore the effectiveness of this treatment approach. This case report reminds clinicians that subgaleal lipomas occasionally present unusually, and a thorough diagnostic workup is essential for appropriate management. Moreover, it emphasizes the importance of meticulous surgical techniques in achieving favourable outcomes. It underscores the significance of vigilant postoperative monitoring to ensure patients' long-term well-being with such rare soft tissue tumours.
